# The Her Tribe and His Tribe Aboriginal-Designed Empowerment Programs

**DOI:** 10.3390/ijerph19042381

**Published:** 2022-02-18

**Authors:** Graham Gee, Sarah Sheridan, Lena Charles, Lana Dayne, Lisa Joyce, Jack Stevens, Yin Paradies, Carol Hulbert, Nick Haslam, Reg Thorpe, Lisa Thorpe, Alister Thorpe, Paul Stewart, Lionel Austin, Louise Lyons, Mary Belfrage, Ruby Warber, Ashley Paxton, Laura Thompson

**Affiliations:** 1 Intergenerational Health Group, Murdoch Children’s Research Institute, Melbourne, VIC 3052, Australia; 2 School of Psychological Sciences, University of Melbourne, Melbourne, VIC 3010, Australia; cah@unimelb.edu.au (C.H.); nhaslam@unimelb.edu.au (N.H.); 3 Clothing The Gaps Foundation, Melbourne, VIC 3056, Australia; sarah@clothingthegaps.com.au (S.S.); lena@clothingthegapsfoundation.org.au (L.C.); laura@clothingthegaps.com.au (L.T.); 4 Victorian Aboriginal Health Service, Melbourne, VIC 3065, Australia; lanadayne@gmail.com (L.D.); rthorpe@vahs.org.au (R.T.); lionel.austin@vahs.org.au (L.A.); 5 Peter MacCallum Cancer Centre, Melbourne, VIC 3000, Australia; lisa_joyce@outlook.com; 6 Murrup Barak, Melbourne Institute for Indigenous Development, The University of Melbourne, Melbourne, VIC 3010, Australia; jack.stevens@unimelb.edu.au; 7 Faculty of Arts and Education, School of Humanities and Social Science, Deakin University, Melbourne, VIC 3125, Australia; yin.paradies@deakin.edu.au; 8 Bubup Wilam Aboriginal Child and Family Centre, Melbourne, VIC 3074, Australia; lisa.thorpe@bubupwilam.org.au; 9 Moondani Balluk Indignenous Academic Unit, Victoria University, Melbourne, VIC 3011, Australia; alisterhenrythorpe@gmail.com; 10 Lowitja Institute, Melbourne, VIC 3053, Australia; paul.stewart@lowitja.org.au; 11 National Indigenous Genomic Program, South Australian Health and Medical Research Institute, Adelaide, SA 5000, Australia; louise.lyons@sahmri.com; 12 The Royal Australian College of General Practitioners, Melbourne, VIC 3002, Australia; mary.belfrage@racgp.org.au; 13 Department of Families, Fairness and Housing Victoria, Melbourne, VIC 3000, Australia; rubywarber@gmail.com; 14 Monash Turner Institute for Brain and Mental Health, Monash University, Melbourne, VIC 3800, Australia; ashley.paxton@fphw.org.au

**Keywords:** Aboriginal and Torres Strait Islander health, Aboriginal community control, program evaluation, mental health, resilience

## Abstract

This study documents evaluation of the Her Tribe and His Tribe Aboriginal-designed empowerment pilot programs. The programs were designed to support Victorian Aboriginal people to strengthen mental health, social and emotional wellbeing, community connection, and to reduce psychological distress. A second aim was to explore participants’ experiences of the programs, including the feasibility and acceptability of the evaluation component. Her Tribe ran for 16 weeks and His Tribe for 12 weeks. In total, 43 women and 26 men completed assessments at pre- and post-program completion, and 17 and 10, respectively, participated in yarning circles at the 6-month follow up. For both programs, there were significant increases in participants’ access to personal strengths and resources, relationship–community–cultural strengths and resources, and decreases in psychological distress. These changes were associated with small to moderate effects that were maintained at the 6-month follow up. There was a significant increase in aerobic fitness for female but not male participants, and no significant changes in weight for either group. Participants described a range of benefits from the programs, including positive elements and areas for improvement. They also viewed the evaluation as feasible and acceptable, and the findings of value. The outcomes from both pilot programs provide evidence that Aboriginal-designed programs, with a focus on physical and cultural activities, can help to strengthen mental health and wellbeing, community connection, and reduce psychological distress in Victorian Aboriginal communities.

## 1. Introduction

In the 2018–19 National Aboriginal and Torres Strait Islander Health Survey, 71 per cent of Aboriginal and Torres Strait Islander people aged 15 years and over reported being in the overweight or obese range, representing a five percent increase from previous national survey data [1,2]. In non-remote areas, 89 per cent of adults did not meet physical activity guidelines for their age [2]. Concurrently, 31 per cent of Aboriginal and Torres Strait Islander adults reported experiencing high or very high psychological distress, and 28 per cent of those living in non-remote areas reported having a mental health condition [3]. Findings of positive relationships between exercise and improved health outcomes are well documented in the international literature. However, extant research does not include an Indigenous population focus [4,5,6]. A population health survey in the United States analysed cross-sectional data that included more than 1.2 million adults and found exercise to be associated with a lower national mental health burden. The strongest associations were identified for participation in team sports (compared to individual sports), with an exercise duration of 45 min and a frequency of three to five times per week [7]. Schuch and colleagues’ [8] meta-analysis of 49 prospective cohort studies that investigated the relationship between physical activity and incidence of depression, included more than 260,000 participants and an average follow up period of 7.4 years. They concluded that people reporting higher levels of physical activity were less likely to report future incidence of depressive episodes across all age groups (youth, adults, and elderly) and geographic locations (Asia, Europe, North America, and Oceania) [8].

Within Australia, there is a limited evidence base supporting the effectiveness of physical activity programs involving Aboriginal and Torres Strait Islander peoples with respect to mental health and wellbeing-related outcomes. A recent systematic scoping review conducted by Macniven and colleagues [9] identified 20 studies that investigated the impact of physical activity and sport on a range of social outcomes among Aboriginal and Torres Strait Islander peoples. The authors noted that the majority of studies utilised qualitative, process evaluation methods, and that most reported positive relationships between physical activity and one of several social outcomes, including education, employment, culture, social and emotional wellbeing, life skills and crime reduction. The authors highlighted the wide range of outcomes and diversity of methodologies employed across the 20 studies, and the need for further development of cultural and social and emotional wellbeing measurement tools [9]. There were few Aboriginal-designed strength-based measures included in these studies that assessed either resilience-related strengths and resources, or Aboriginal mental health-related difficulties such as depression or psychological distress.

To address the need for strengthening mental health and social and emotional wellbeing, and reducing psychological distress and the risk of chronic disease within the Victorian Aboriginal community, healthy lifestyle manager Laura Thompson (co-author) and her team from the Victorian Aboriginal Health Service (VAHS) in Melbourne, Australia, developed the Her Tribe and His Tribe pilot programs. Her Tribe, developed in 2017, was a 16-week program for Aboriginal and Torres Strait Islander women. His Tribe, a 12-week program for Aboriginal and Torres Strait Islander men, was developed in 2018. Both programs were designed with a strong community health promotion and chronic disease prevention focus. The present study is an evaluation of the two programs.

The first aim of the evaluation was to investigate pre- and post- program changes for participating women and men, across multiple physical and social and emotional wellbeing domains. The social and emotional domains included personal, relationship, community and cultural strengths and resources. These strengths and resources were assessed using the Aboriginal Resilience and Recovery Questionnaire (ARRQ) [10], an Aboriginal-designed strength-based measure, developed at VAHS, that was being used for the first time in a community program setting. Individual assessments using the ARRQ in this study are underpinned by two important understandings of resilience held by the co-authors and program staff. First, it is a concept grounded in culture, linked to histories of ancestral and cultural connections that precede colonisation [11], in addition to recognising the ongoing impacts of colonisation. It reflects processes by which individuals, families, communities, and whole nations overcome adversity, and this may involve resistance, justice, restoration, and processes of accessing and renewing personal, family, community, and cultural strengths and resources. It is, therefore, a multi-dimensional construct that encompasses personal strengths or qualities, the capacity to utilise a repertoire of behavioural skills (i.e., action-based coping strategies), as well as access to supportive relationships and systems-level, structural resources. Accordingly, the terms ‘strengths’, ‘resources’ and ‘skills’ are used throughout the paper to emphasise different aspects of resilience. We note that these understandings align with other alternative understandings of resilience that place the concept within contextual and ecological frameworks [12].

Additional evaluation measures included changes in participants’ levels of psychological distress, aerobic fitness and weight. Based on the extant research, initial hypotheses predicted significant increases in women and men’s access to strengths and resources, significant reductions in psychological distress, significant increases in women and men’s aerobic capacity and significant decreases in body weight. The second aim was to investigate participants’ qualitative feedback about the programs, including perceived benefits of the program for participants, areas for improvement, and participants’ overall experience with the research component of the programs.

## 2. Materials and Methods

### 2.1. Participants

From October 2016 to January 2017, and January to March 2018, the VAHS Healthy Lifestyle unit used its Facebook, Instagram and other social media platforms to recruit female and male members of the Melbourne Aboriginal community to participate in the Her Tribe and His Tribe programs. The programs were also advertised and promoted by staff for community members using the Family Counselling Services of VAHS. Hence, composition of the participants is best viewed as Aboriginal and Torres Strait Islander people within the Melbourne community who access Aboriginal Community Controlled Health services, rather than being representative of the whole Victorian Aboriginal and Torres Strait Islander population. In addition, as the primary function of VAHS is service delivery, maximum community participation and program accessibility was a priority. Therefore, community members were invited to participate at any stage of the programs. Engagement in the research evaluation component of the program was viewed as a secondary goal, as the team did not want to discourage participants from receiving the benefits of the program due to concerns about the research component.

### 2.2. Program Design

A single group, pre- and post-test study design was used for both programs to assess short-term outcomes, followed by a 6-month post-program assessment that included qualitative yarning circles ( ‘yarning’ groups or circles is a term used by some Aboriginal and Torres Strait Islander communities to refer to processes of bringing a select group of community members together for the purpose of gathering specific information, in accordance with local community and cultural protocols. In a research context, yarning circles share some congruency with focus groups [13]). Prior to Week 1, interested community members attended an information evening that included presentations about the program aims and activities, and an invitation to participate in the research and evaluation component (this study). For both programs, participants were invited to attend a weekly two and a half hour evening group session at a local Aboriginal community-controlled organisation. Sessions commenced with sharing a meal, followed by an inspirational presentation by a majority of Aboriginal speakers. Most of the speakers were either well-known local Aboriginal community leaders, or well-known national Aboriginal figures. Each speaker shared stories about their personal successes and experiences of reaching goals and overcoming adversity. The talks were approximately 45 min in length and included question and answer time. Table 1 lists the weekly topics and session themes, and a broad description of speaker content. For confidentiality purposes, the names of speakers have not been listed. Following the presentations, participants trained together for one hour, engaging in physical activities that were led by personal trainers. Activities varied each week, ranging from circuits and weights to group competitions and cultural games. Participants were provided with additional opportunities to engage in a group activity each weekend. Weekend activities included hiking, playing cricket, roller skating, and attending a football game. The programs culminated in a weekend camp and group celebration. Three changes were made to the His Tribe program as a result of Her Tribe program feedback. This included introducing a cultural smoking ceremony at the beginning of each weekly session, introducing two Aboriginal personal trainers to lead the program activities, and having the His Tribe program run for 12 rather than 16 weeks due to funding and resource constraints.

### 2.3. Outcome Measures

Aboriginal Resilience and Recovery Questionnaire (ARRQ): The ARRQ is a 60-item multidimensional strengths questionnaire that was designed to assess strengths and resources associated with resilience, healing and recovery among Aboriginal and Torres Strait Islander help-seeking populations. The ARRQ uses a 5-point Likert scale response format with ratings from 1 = Not at all to 5 = A lot, and it includes a range of strength-based constructs and sub-scales (2–5 items), such as the following: community connection, cultural identity, self-worth, emotion regulation, strong relationships, safety, a personal sense of mastery, spirituality as a source of strength, and participation in cultural practices. The measure comprises several single items, and two major subscales that represent personal strengths (29 items) and relationship–community–cultural strengths (21 items). The two major subscales can be summed to yield a composite total strengths score. Both subscales and the total strength scores of the ARRQ have previously demonstrated high internal consistency, with a Cronbach’s alpha value of 0.89 for the personal strengths component, 0.88 for the relationship–community–cultural strengths component, and 0.93 for the total strengths composite measure of the AARQ [10].

Kessler Psychological Distress Scale (K10): The K10 [14] is a 10-item screening tool used to measure non-specific symptoms of psychological distress during the past 30 days (e.g., feeling hopeless, nervous, restless, worthless, tired for no reason, sad). The K10 uses a 5-point Likert scale rating from 1 = None of the time to 5 = All of the time, and yields a score between 10–50, with well-established symptomatic cut-off points to describe severity of psychological distress (see Appendix A for the 10 screening items). Scores of 30 and above indicate very high levels of psychological distress and a likelihood of significant mental health difficulties. The K10 has been used in Aboriginal and Torres Strait Islander health-related surveys, including the 2008 Victorian Population Health Survey and in research investigating the relationships between psychological distress, stressful life events, and adverse health outcomes among Victorian Aboriginal and Torres Strait Islander peoples [15].

Body Weight: Participant body weight was measured at baseline (week one) and final week of the programs (week 16 and 12) using electronic scales.

Aerobic capacity: Aerobic capacity was measured at baseline (week one) and final week of the programs (week 16 and 12) using the 20 metre Multistage Fitness Test (Beep Test) [16].

### 2.4. Yarning Circles

Participants were invited to be involved in yarning circles at six months post-program. Three audio-recorded yarning circles for 17 Her Tribe participants and one for 10 His Tribe participants were conducted using a semi-structured group interview format. Participants were asked five questions during the yarning circles, which included the following: (1) What was your experience of the program and why did you keep coming back? (or why did you not?) (2) What (if any) changes did you experience and has that continued? (3) How could we improve the program? (4) How did you feel about it being a research project? (5) Is there anything that we have not asked about that you would like to add?

### 2.5. Safety and Wellbeing Protocol

A safety and wellbeing protocol was developed that included multiple wellbeing check-in processes to engage any participants who reported significantly high levels of psychological distress during the program and might require support. First, an Aboriginal psychologist from VAHS attended all sessions to provide participants an opportunity for support if required. Second, following the weekly evening sessions, the psychologist and designated team member met to discuss whether there were any participants who had approached the team for support, and/or whether the team had observed any participants who appeared to require support. Third, during the assessment weeks (week 1 baseline, post-program week 16 for Her Tribe and week 12 for His Tribe, and 3 month follow up week for both programs), the designated team member and the Aboriginal psychologist reviewed participant scores on the K10 measure of psychological distress. If a participant scored in the very high range (>30), they were contacted. The initial call comprised a ‘wellbeing check’ to let the participant know about the high psychological distress score and a general enquiry about their current wellbeing and whether they needed any support(s). The option for formal support was provided through referral to either a VAHS Aboriginal psychologist or two other psychologists external to the organisation that were available throughout the duration of the programs.

### 2.6. Data Analysis

Descriptive statistics were conducted to examine demographic characteristics of participants from both programs. The IBM statistical software package SPSS Version 22 [17] was used to conduct dependent t-tests to assess pre- and post-program changes in participant-reported access to personal strengths and resources, relationship–community–cultural strengths and resources, psychological distress, aerobic fitness and weight. Cohen’s *d* was used to calculate the measure of effect size, with 0.20, 0.50 and 0.80 representing small, moderate, and large effect sizes [18]. To assess for the maintenance of changes in psychological distress and access to strengths and resources, dependent t-tests also compared scores at week of program completion and 6-month follow up.

For the yarning circles, thematic analysis [19] was used to investigate participants’ experiences of the programs. The yarning circles were audio-recorded, transcribed and independently hand-coded by one non-Aboriginal and two Aboriginal team members (co-authors LD, GG, and LT). Co-author LD conducted an initial open, inductive coding round of all transcripts and then the three team members met to review and discuss the initial codings. Co-authors GG and LT then independently analysed all transcripts and the three co-authors met to further refine codings and identify common categories. Comparative coding continued until saturation was reached. An iterative process of coding was used, beginning with ‘open coding’ of broad categories, ‘axial coding’ where codes were more narrowly specified, and ‘selective coding’, where codes were arranged according to sub-themes and core themes [20].

### 2.7. Ethics and Methodology

The study was reviewed and approved by the VAHS research subcommittee and the University of Melbourne Human Research Ethics Committee in 2016 (Ethics ID 1648120.1). The methodology that underpinned the study followed Rigney’s [21,22]) Indigenous research principles, with Aboriginal voices privileged throughout the design, implementation, and evaluation of the programs. The ethos of self-determination was integral to the program and evaluation component in that it was designed and led by Aboriginal health professionals. For example, Aboriginal staff were employed to run the program; a reference group was established that included Aboriginal elders and community leaders; the project was endorsed by the VAHS Board of Directors; all research participants from both programs were Aboriginal community members; the evaluation was led by Aboriginal researchers; and an Aboriginal-designed, strength-based assessment tool was used in both programs.

## 3. Results

In total, 120 women and 37 men attended at least one session of the programs. Of those, 86 women and 31 men elected to participate in the evaluation study of the program, with some joining after the baseline assessment. The week one baseline and post program assessments were completed by 43 women and 26 men. The demographic characteristics of all participants who elected to complete at least one assessment are presented in Table 2. Forty-three women and 26 men completed the pre- and post-program assessments that included the ARRQ and psychological distress, while 37 women and 26 men completed the post-program and 6-month follow up. The completion rate for biometric measures of weight and aerobic capacity were significantly lower and, therefore, only the pre- and post-program assessments were conducted. A total of 18 women and 6 men completed pre-and post-program aerobic assessments for aerobic capacity, and 14 women and 14 men for weight.

**Figure 1:**
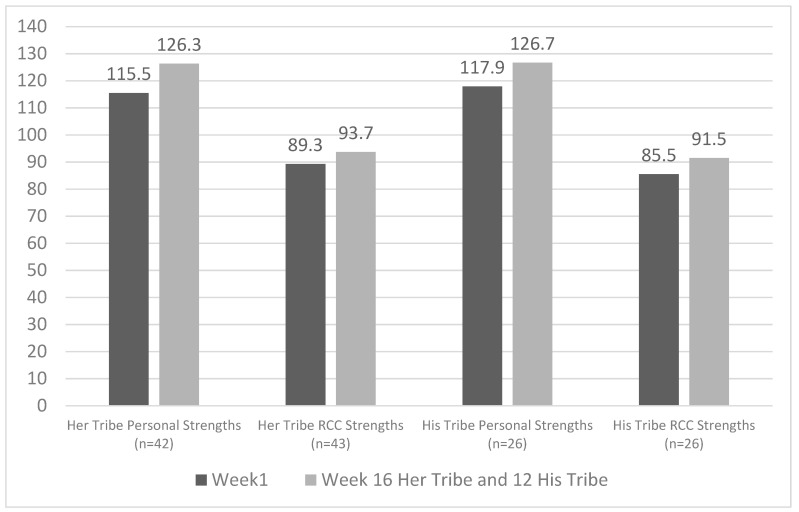
Significant increases in access to Personal Strengths and Relationship–community–cultural (RCC) strengths for participants from the Her Tribe and His Tribe pilot programs.

**Figure 2:**
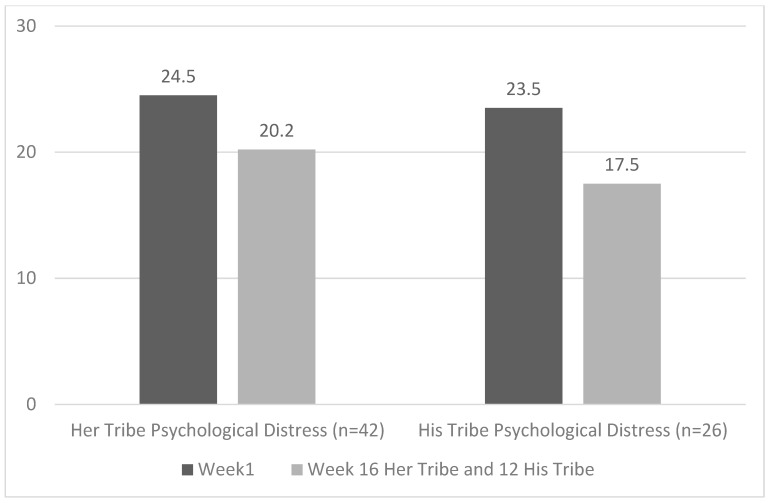
Significant reductions in psychological distress for participants from the Her Tribe and His Tribe pilot programs.

Descriptive statistics of pre-and post-program, and 6-month follow up outcomes are presented in Table 3, Table 4, Table 5 and Table 6. Statistical analyses identified significant increases in women and men’s reported access to personal strength and resources, and relationship–community–cultural strengths and resources, from pre- to post-program assessment, with the effect sizes for these associations ranging from small to moderate. Also, for both men and women, a significant decrease in psychological distress from pre- to post-program assessment was found, with associated moderate effect sizes. All changes were maintained at 6-month follow up. The pre- to post-program changes in personal strength and resources, relationship–community–cultural strengths and resources, and psychological distress for the two programs are illustrated in Figure 1 and Figure 2. There was a significant increase in aerobic fitness for female but not male participants, and no significant changes in weight for either group.

### 3.1. Yarning Circles

**Figure 3:**
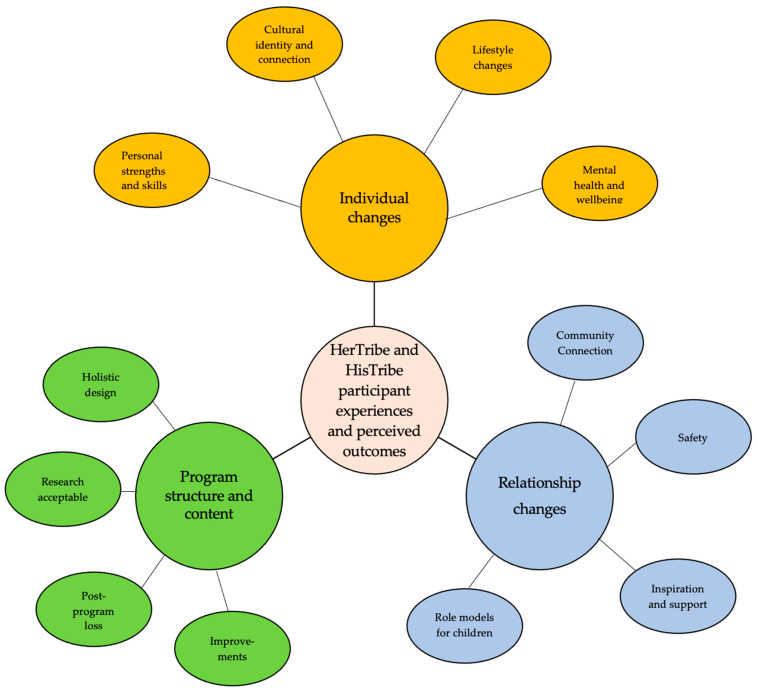
Participants’ program experiences and perceived outcomes.

Independent coding of the four yarning circles, held between 2017–2018 by authors LT, LD and GG, resulted in an initial set of 40 open-coded, broad categories. The axial coding phase involved re-reading transcripts and the three authors meeting to discuss and explore new connections and relationships between codes. This iterative process of comparison and differentiation resulted in narrowing to 23 axial codes. These codes were then utilized as a primary coding framework. In the final phase of selective coding, two important processes influenced the generation of sub-themes and core themes. First, the codes were examined with the five yarning circle research questions in mind, and this began to frame themes according to participants’ experiences and impacts of the program, and program content and structure. Second, researcher reflexivity was an important part of the selective process, as all three researchers’ cultural and professional backgrounds were recognized as influencing interpretation of participants’ experiences of the program. For example, the influence of lead program director LT’s extensive experience in health promotion helped shape discussions that resulted in generating sub-themes and recognizing connections between program improvement and post-program loss. The extensive counseling background of researcher GG, in working with community mental health and developing Aboriginal-designed assessment tools, informed discussion about exploring connections and codes associated with mental health. The non-Aboriginal background of researcher LD provided informed contrasts and differences in worldviews, and her key program involvement in all participant assessments and co-facilitation of yarning groups provided reflections and discussion based on her in-depth immersion with participants. Taken together, this provided a rich diversity of perspectives, with regards to reaching consensus around removing codes with not enough supporting data and generating sub-themes and core themes. The axial codes were reduced to 12 subthemes, thematically arranged according to three core overarching themes. The three core themes are titled ‘Individual changes’, ‘Relationship changes’, and ‘Program Structure and Content’, each with four associated subtheme areas. Figure 3 is a thematic map of the participants’ experiences and perceived outcomes of the programs showing the core themes and associated subthemes.

The core theme ‘Individual changes’ represents personal experiences shared by participants from both programs that included a wide range of changes, related to personal strengths and skills (subtheme), and changes in lifestyle choices and behaviours (subtheme). The types of personal strengths, skills and benefits frequently identified across both programs included feelings of positivity (‘I kept coming back because I loved it’), and increased confidence and self-belief (‘It gave you belief in yourself’), as a result of being challenged to push past one’s comfort zone (‘There was a lot of obstacles, things that we probably thought we’d never be able to get through or achieve’). The types of lifestyle changes discussed by participants focused on areas of life such as diet and exercise (‘My eating habits have changed’, ‘I maintained sort of a fitness regime after the program’), as well as changes in self-care strategies. For example, one participant spoke about changing her capacity for self-care by expanding her range of strategies, stating ‘‘I find other ways to, I do other things, I’ll take the dog for a walk… or just take time out to myself…actually make time for myself now, whereas I never used to, it used to always just be work, go home, deal with the family and then pretty much go to bed and then repeat every day.’ A third, and among the most prominent, subthemes identified by participants, was the notable benefits in mental health (‘It was because of Her Tribe that my head changed…. I was able to look to the future’). Participants also spoke about the programs as a culturally safe space that fostered cultural connection and cultural identity (subtheme) (‘When a group of Aboriginal men come together, it makes it more cultural, it makes them want to join in more’, ‘That’s what made it so important, belonging’).

‘Relationship changes’, as a core theme, referred to several different dimensions of participant relationships that changed throughout the programs. Possibly the strongest qualitative subtheme to emerge in all yarning circles was the overwhelming number of participants that described experiencing a sense of strengthened community connection. For some participants, this was seen within contexts of past adversity (‘When you don’t really have a sense of a great family or you have issues within your family… you know there’s community out there… it’s really taken that isolation level off’) and more broadly, connection in general (‘It’s great just to connect with people that you wouldn’t have known otherwise’, ‘Just the people, the connectedness’). Importantly, the role of strengthening connection appeared to function in ways beyond simply increasing the number of supports and connections that participants experienced. For example, community connections helped one participant with grief and loss (‘It did relieve the stress, I come one day after me brother’s funeral and that and it was the best place I could have been. And people said that I shouldn’t have gone but I, I wanted to be with the group’). For other participants, the increased community connection and support helped to validate their roles as parents, as seen below:

*‘We all connected as mothers or as women, but we all had the same issues and just being in a place that it’s okay to feel like that because everybody has been through that moment or felt like that before or could understand I could just talk to somebody even if it was an aunty or another women that has a young child the same age. I could just get some comfort to say, it is okay and I’m doing a good job’*.

(Yarning Group 3 Participant, Her Tribe)

Participants also identified safety (subtheme) in the environment as being important (‘To me it was just a very safe environment where people could be whoever, just be themselves’), and the opportunity to be role models (subtheme) for children who were welcomed to attend (‘It helps them as well to see that if mum…is doing something to stay healthy…. then they can learn from that’). The last relationship subtheme was that of receiving inspiration and support from others, both in terms of other participants and the speakers that shared personal stories at the beginning of each weekly session (‘Everyone bought their story and their strength and their challenges and their strategies and you learnt from that’, ‘Every week you’d have a different speaker and they’d motivate you in something different’).

The core theme, titled ‘Program structure and content’, contains several distinct subthemes. Positive feedback provided by participants included the holistic design of the program (subtheme), notably the inclusiveness of inviting whole families and people of all ages from the community to attend (‘I really liked it because it, it catered for all ages like Elders and young people and you could bring your kids’). Participants also identified the value of having speakers share personal stories of success and overcoming adversity, the variety of weekly activities rather than a set routine, and the end of program camps were widely praised as a highlight (‘I loved the camp. The camp is probably where I had my ‘ah-hah moment’).

Limitations and improvements (subthemes) for future programs were also identified by participants. For example, the set weekly evening time was seen as a positive by many participants because it allowed working community members to attend and bring their children. However, this timeslot posed challenges for some families. While healthy meals were provided during the weekly session, this didn’t necessarily equate to a full family meal, and parents described the challenge of needing to try and prepare children’s meals before the program or having to ‘quickly grab something on the way home’, which didn’t necessarily align with the healthy foods focus of the program. Transportation was a challenge for some families that limited regular attendance at the weekly sessions. Some improvements suggested by participants included switching the order of activities, for example not sitting down for a shared meal before the evening speaker, and instead engaging in the training and physical activities first. Other suggestions for improvement included introducing additional cultural activities, such as basket weaving and cultural dance, and also having a camp at the beginning of the program.

A notable subtheme that emerged across all yarning circles was experiencing a sense of loss following the program completion. Some participants reported losing momentum and regularity of training. The most common loss described by participants was a loss of connection and group support (‘Ok, so eventually stopping the program for me really put me on a downer…there’s no connection like we had’), and this prompted participants to query whether scaled-down versions of the programs could be run in the future.

The final subtheme related to ‘Program structure and content’ reflects the consistent feedback from participants that the evaluation component of the program was acceptable and that the findings were of value. Some participants spoke about an initial reluctance (‘At the start I was a bit like, not iffy, but it was a bit like “oh ok, so what parts and all that?”). However, as the program progressed, participants shared that they developed a better understanding of the purpose of the research, and they valued seeing personal changes after filling out the questionnaires (‘It’s interesting to see how, how you actually have changed over the period of time. It’s good to see the progress’). Reflections on the use of the Aboriginal Resilience and Recovery Questionnaire (ARRQ) that was designed at the Victorian Aboriginal Health Service were important to capture in the evaluation, as it was the first time the tool had been used by the organisation within a service delivery program context to assess pre- and post- changes among community members. Positive feedback was received in the yarning circles, as well as in a feedback section added to the paper form of the ARRQ to gather information about participants’ experience of filling it out. Table 7 and Table 8 list examples of participant quotes from both programs that illustrate each respective subtheme, including use of the ARRQ.

### 3.2. Post Hoc Analysis

Experiences of feeling safe, a strengthened sense of community connection, and positive mental health and wellbeing were all prominent themes shared by participants from the yarning circles. This is consistent with the quantitative results that demonstrated reductions in psychological distress and increases in access to personal strengths and relationship–community–cultural strengths. These two ARRQ subscales include several smaller subscales (i.e., 2–5 items), specific to safety, community connection, positive emotions and self-worth (i.e., aspects of positive mental health). As a way of triangulating findings from the yarning circle subthemes, a series of post-hoc independent sample t-tests were conducted to explore potential pre-and post-changes for both programs, for the safety, community connection, positive emotions, and self-worth ARRQ subscales. As illustrated in Table 9 and Table 10, there were significant increases from pre- and post-assessment in all of these subscales for the Her Tribe and His Tribe programs.

## 4. Discussion

The Her Tribe and His Tribe Aboriginal-designed empowerment programs were innovative pilot programs initiated by the Victorian Aboriginal Health Service (VAHS) healthy lifestyle team, with a focus of strengthening wellbeing and reducing the risk of chronic disease within the Melbourne Aboriginal and Torres Strait Islander community. Both programs aimed to support community members that were connected VAHS (i.e., service users and/or community members linked to the organisation via social media platforms), to increase their capacity to access a range of strengths and resources associated with positive mental health and social and emotional wellbeing, reduce psychological distress, and increase aerobic fitness and decrease weight. The programs provided a secondary opportunity for VAHS to implement a program evaluation, which included utilising and further validating the Aboriginal Resilience and Recovery Questionnaire (ARRQ) and conducting yarning circles to better understand community members’ experiences of the programs.

The reductions in psychological distress that were maintained at the 6-month follow up are an important finding given that Victorian Aboriginal and Torres Strait Islander people report elevated rates of psychological distress in comparison to other Victorians [3]. It suggests that these programs can help to strengthen the mental health of Victorian Aboriginal and Torres Strait Islander community members. To our knowledge, few, if any, evaluations of Aboriginal and Torres Strait Islander exercise-focused programs have documented short to medium-term changes in mental health using quantitative measures and, accordingly, this study helps to build on current evidence for the benefits of these types of programs [9]. The mental health benefits of both programs are further supported by the finding that participants reported an increase in access to a wide range of personal, relationship, community and cultural strengths and resources 6-months post-program. These same strengths were associated with lower post-traumatic stress and depression symptom severity, and higher levels of empowerment, among Aboriginal help-seeking clients using the VAHS counselling service in 2011–2012 [10].

A third positive impact for the program, from a mental health perspective, was that feedback from participants (and staff) from both programs about the opportunity for support via referrals to an Aboriginal psychologist, or psychologists well-linked to the community-was viewed as a positive initiative. Twenty-eight participants from the programs reported very high psychological distress at least once during the program, and of those, 10 elected to utilise one of the psychologists (six participants from the Her Tribe program and four from the His Tribe program). Anecdotal feedback from these participants was that the opportunity for counselling had been a valuable experience and that the offer of support had been appreciated. Of the participants who declined any formal support, reasons given included that they were already seeing a counsellor or psychologist and had adequate support; that despite the elevated score they were doing well enough not to need support; or, they had experienced a stressful week during the time of filling out the questionnaire but were doing well since then. We suggest that offering opportunities for mental health support in programs such as these can help to normalize and de-stigmatise the process of reaching out to others and seeking help. It would be of benefit in future programs to systematically investigate participants perspectives about whether having explicit support processes in place can help to decrease stigma associated with experiencing mental health and social and emotional wellbeing difficulties.

There was a significant increase in aerobic capacity for female, but not male, participants. One possible reason for this differing result is that the Her Tribe program was four weeks longer in duration and provided a greater frequency of training and time of exposure to exercise. Another is that the number of men who completed the pre- and post-program aerobic assessment was low. Injuries and physical disabilities contributed to only six of 34 male participants completing both assessments, and such a low number resulted in an underpowered t-test analysis. It is worth noting, however, that the number of female participants who completed pre- and post-program aerobic assessments was also low, with only 18 of 43 participants completing both. The small participant numbers mean these findings need to be interpreted with caution, and the poor completion rates suggest that the multistage fitness test may not be the most appropriate form of aerobic assessment for future programs.

There were no significant changes in weight for either group at post-program assessment. It should be noted that this was not the primary goal of either program, as the emphasis for both programs was on wellbeing, increasing personal agency, and healthy lifestyle changes. One plausible explanation is that one hour of exercise a week was not enough exposure to physical activity, from an intervention design perspective, to expect significant reductions in weight. For example, Canuto and colleagues [23] reported moderate reductions in weight and body mass index for 100 Aboriginal and Torres Strait Islander women from the Adelaide metropolitan region, participating in a 12-week exercise and nutrition program. However, their program included two 60-min group cardiovascular and resistance training classes per week.

Data from the yarning circles suggested the men and women experienced a wide range of social and emotional wellbeing benefits from the programs. This finding is consistent with those of a scoping review that found evidence for relationships between physical activity programs involving Aboriginal and Torres Strait Islander people, and social outcomes that included strengthened identity, community connection, and reconnection to culture [9]. Our findings are also congruent with a previous study that used semi-structured interviews and focus groups with young Aboriginal men from the same Aboriginal Melbourne community as our study. Thorpe, Anders, and Rowley [24] explored the impacts of an Aboriginal community sporting team on the social, emotional, and physical wellbeing of young Aboriginal men. The key themes that emerged included categories such as community connection, cultural values and identity, and sub-concepts that referred to building safety, self-esteem, and enjoyment, while reducing players’ stress, sense of isolation, anxiety and fears (e.g., psychological distress). These themes are strikingly similar to those identified in this study, and as described in the results section, these themes reflected participant experiences of belonging and shared culture. We believe this is a particularly salient point, given that there was, in fact, a large cultural diversity of Aboriginal and Torres Strait Islander peoples participating in the programs - with more than 20 different clan group affiliations being reported in the demographic part of the program sign up questionnaire. At the same time, the Victorian Aboriginal Health Service (VAHS) is located within Metropolitan Melbourne, an urban geographical location, where Aboriginal and Torres Strait Islander people are a minority group in population size. As such, we believe these programs, and VAHS as a cultural institution, are spaces that signify cultural anchors in urban environments that can otherwise undermine the visibility and representation of Aboriginal and Torres Strait Islander peoples. Taken together, these findings further strengthen the evidence base that for the Melbourne Aboriginal community, programs and activities with a physical and cultural focus can greatly contribute to strengthening cultural and community connection, positive mental health, and social and emotional wellbeing.

The yarning circle theme of ‘Program structure and content’ included participants’ thoughts about ways to improve the programs. A common suggestion for program improvement was to try and find a way to extend the duration of the programs, and this was directly linked to many participants experiencing a sense of loss after the programs had ended. This raises important issues about how programs, such as Her Tribe and His Tribe, can be funded and resourced in a sustainable way that allows for program longevity. That is, how to embed access to health and wellbeing programs for community members on an ongoing basis. The yarning circles and discussion among VAHS staff included ideas of paring or stripping back the contents and structure of the programs, to potentially minimise the amount of staffing and resourcing required for running such programs. Exactly which areas of program content and delivery are essential, and which areas could be pared back, requires further investigation. Future research could involve investigating the ‘core elements’ of success for program implementation, from both participant and staff perspectives, as a way to adapt program design and funding needs, and achieve sustainability and program translation.

Implementation of a research and evaluation component for both programs was viewed as acceptable and feasible by participating community members, and the findings of value. The ‘how’ and ‘why’ the research component was deemed acceptable and valued is important to consider. We suggest that it was largely due to the level of input and contribution from Aboriginal community members. For example, the programs were designed by an Aboriginal manager and the VAHS healthy lifestyle team, and the evaluation was led by an Aboriginal psychologist, who worked at VAHS and developed the ARRQ during that time. Key Aboriginal Elders and leaders from the community were involved at different levels (from the VAHS Board of Directors, to the research committee, to the established reference group), all of whom played a role in overseeing how the program was implemented. Of relevance to this level of local Aboriginal engagement, we note that 15 years ago a series of Victorian Aboriginal community wide consultations about research were conducted, resulting in one report titled ‘We can like research…. In Koori Hands’ [25]. One of the recorded sessions was titled ‘What we expect from Koori Health Research by 2020′, and among important expectations were listed ‘strong community approach to Koori health research; increase in research capacity and more students doing PhDs; more control over health and research direction; and better health and social outcomes’ (p. 42). Arguably, the design and implementation of the Her Tribe and His Tribe programs, including the research and evaluation component, integrated these priorities. Perhaps not surprisingly, four of the forty community members who attended the 2007 workshops were key members, involved in either overseeing or implementing these programs (all co-authors).

There are several limitations in the study design that need to be acknowledged. For example, whilst community engagement at any stage of both programs was a service delivery priority, such flexibility in program participation meant that attendance rates varied throughout the programs, and it was difficult to measure dose–response effects for either program. Community members’ prior mental health vulnerabilities were also not assessed, nor was nutritional/energy intake throughout the programs. Both of these factors are potential confounding factors that could have influenced changes reported by participants during the programs. We also acknowledge that there is a likely selection bias in that participants recruited were already engaged with VAHS, either as direct service users or connected to VAHS via its’ social media platforms. Although we are confident that the participants represent a broad and diverse part of the Victorian Aboriginal and Torres Strait Islander community, we cannot claim that they are representative of the respective general population. Finally, the number of participants was relatively low, which resulted in reduced statistical power for the quantitative analyses.

These limitations notwithstanding, there are some strengths that underpin the study.

Perhaps most importantly, both programs and the research were designed and led by Aboriginal and Torres Strait Islander people/staff from the community, and this contributes to the self-determination and empowerment of the community. Another strength of the study is the utilisation of the ARRQ, an Aboriginal-designed measure that was developed at VAHS for service use in the local Victorian Aboriginal and Torres Strait Islander community. The ARRQ had previously demonstrated content, discriminant, and predictive validity, with regards to lower mental health difficulties (i.e., trauma and depression symptom severity) [9]. Now it has been used in a community program setting, with demonstrated sensitivity to change with regards to the wide range of personal, relationship, community and cultural strengths that comprise the measure.

## 5. Conclusions

The Her Tribe and His Tribe programs involved Aboriginal leadership at all levels of design and implementation, and the research and evaluation component of the programs alone were accessed by over 100 community members. Despite the limitations of the study, the multi-method evaluation showed clear association with, and/or evidence of, increased personal, relationship, community and cultural strengths and resources, reduced psychological distress, and increased community connection among participating community members. When led by Aboriginal researchers and services, it is possible to utilise Aboriginal research methodologies and combine qualitative and quantitative evaluation methods that include the use of validated, Aboriginal-designed tools, to demonstrate the benefits of Aboriginal-designed health and empowerment programs.

## Figures and Tables

**Table 1:** Her Tribe and His Tribe Weekly program topics, themes for sessions and speakers.

	Her Tribe Program			His Tribe Program		
Week	Topic	Themes	Speaker	Topic	Themes	Speaker
1	Getting in to your groove	Team Bonding Fitness Test	Program leader	Fitness…The Black Way!	Team Bonding Fitness Testing	Aboriginal fitness promotion
2	Today’s Warriors	Resilience Motivation	Kokoda Trek Leader	Talking Nutrition	Diet and lifestyle	Nutritionist AFL Players
3	It’s Never Too Late	Determination Overcoming challenges Goal Setting	Aboriginal Ultra Marathon Runner	Tackling the Beast	Resilience Suicide prevention	Aboriginal mental health
4	Destination Arnold	Goal setting Determination Inspiration	Aboriginal Body Builder	The Premiership Race	Exercise promotion Diet	Aboriginal AFL Players
5	Eagle, Look out!	Community Building Wellbeing	No speaker	Soaring with Bunjil	Physical/mental barriers Connecting with country	No speaker
6	Receiving the Gold	Resilience Commitment	Aboriginal psychologist	You are in Control	Gambling prevention Fitness testing	Gambling prevention speaker
7	Tough Mudder Champion	Fitness testing Overcoming Challenges	Tough Mudder Competitor	Over the Hurdles	Coming togetherTeamwork	Aboriginal Psychologist
8	Women’s Circles	Peer leadership Self-Esteem	Aboriginal domestic violence survivor and educator	Receiving the Gold	Self determination	Aboriginal boxer and fitness trainer
9	Her Time–Caring for yourself	Yoga Self-Care	Aboriginal Yoga Teacher	Sporting Stars	Northcote Sporting Competition	No speaker
10	I am not the problem	Self-Determination Leadership	Aboriginal Community Leader	Your Inner Ninja *	Health and fitness choices Overcoming adversity	‘Deadly Ninja’ success
11	Chasing Goals Balancing Family	Fitness testing Goal Setting self and family wellbeing	Aboriginal Jui-Jitsu Champion			
12	First 1000 Days	Education Women’s Health	Aboriginal academic leader			
13	Throwback Thursday	Northcote Sporting Competition	No speaker			
14	Women’s Circle	Community care Relationships Connectedness	Aboriginal Woman and community champion			
15	Breaking Ground	Trauma Resilience Culture	Aboriginal community champion			
16	Graduation	Fitness Testing	No speaker			

* The theme ‘Inner Ninja’ is a reference to the television series ‘Australian Ninja Warrior’ as that week involved a past Aboriginal participant from the series describing their experiences and training for the program.

**Table 2:** Participant Demographics.

	Her Tribe Participants (*n* = 86)		His Tribe Participants (*n* = 31)	
	Total	Percentage *	Total	Percentage
Indigenous status/affiliation				
Aboriginal	74	86.0	31	100
Torres Strait Islander	1	1.2	0	0
Aboriginal and Torres Strait Islander	2	2.3	0	0
Bi-cultural heritage	3	3.5	0	0
Identified as Koori (clan/language group within Victoria)	47	58.8	16	51.6
Non-Koori (identified clan/language group outside Victoria or did not answer)	33	41.3	15	48.4
Age (years), Mean (SD)	35.7 (SD 12.1)		40.3 (SD 13.0)	
Employment				
Yes	61	70.9	23	74.2
No	19	22.1	8	25.8
Financial security Enough money for basic living expenses				
Yes	72	83.7	27	87.1
No	8	9.3	4	12.9
Education **				
Completed year 7–11	38	44.0	18	58.1
Completed year 12	42	49.0	13	41.9
Completed a tertiary degree	26	30.0	9	29.0

* Percentage calculations do not add to 100 per cent due to participant incompletion of demographic data. ** Education totals and percentages exceed sample size and 100 per cent due to some participants not completing year 12 also reporting going on to complete further studies that resulted in obtaining a tertiary degree.

**Table 3:** Her Tribe Participant Personal Strengths, Relationship–community–cultural Strengths (RCC), Psychological Distress, Aerobic capacity and Weight outcomes at Pre- and Post-Program Assessment.

Outcomes	Her Tribe Pre- and Post-Program Comparisons					
	Pre M (SD)	Post M (SD)	Mean Diff	95% CI	t	Cohen’s d
Personal Strengths (*n* = 42)	115.52 (17.13)	126.26 (17.40)	10.74	5.77, 15.70	4.37 ***	0.67
RRC Strengths (*n* = 43)	89.30 (13.04)	93.67 (12.07)	4.37	1.00, 7.73	2.62 *	0.40
Psychological Distress (*n* = 42)	24.50 (8.10)	20.19 (7.21)	4.31	−6.48, −2.14	−4.00 ***	−0.61
Aerobic (*n* = 18)	3.94 (0.61)	5.76 (0.77)	1.81	1.15, 2.49	5.89 ***	1.53
Weight (*n* = 14)	73.26 (16.72)	71.97 (16.23)	1.29	−2.82, 0.24	1.78	−0.41

* *p* < 0.05, ** *p* < 0.01, *** *p* < 0.001.

**Table 4:** Her Tribe Participant Personal Strengths, Relationship–community–cultural (RCC) Strengths, and Psychological Distress outcomes at Post-Program and 6-month Follow up.

Outcomes	Her Tribe Post-Program and 6-Month Follow up Comparisons					
	Post *M* (SD)	Follow up *M* (SD)	Mean Diff	95% CI	t	Cohen’s d
Personal Strengths (*n* = 37)	125.05 (17.71)	125.08 (14.83)	0.03	−3.61, 3.66	0.02	0.00
RCC Strengths (*n* = 37)	93.43 (12.55)	94.05 (9.62)	0.62	−1.98, 3.22	0.48	0.08
Psychological Distress (*n* = 36)	20.81 (8.37)	20.36 (8.62)	0.44	−2.50, 1.61	−0.44	−0.07

* *p* < 0.05, ** *p* < 0.01, *** *p* < 0.001.

**Table 5:** His Tribe Participant Personal Strengths, Relationship–community–cultural (RCC) Strengths), Psychological Distress, Aerobic capacity and Weight outcomes at Pre-and Post-Program Assessment.

	His Tribe Pre- and Post-Program Comparison					
	Pre *M* (SD)	Post *M* (SD)	Mean Diff	95% CI	t	Cohen’s d
Personal Strengths (*n* = 26)	117.85 (14.32)	126.66 (13.72)	8.81	3.76, 13.86	3.59 ***	0.69
RCC Strengths (*n* = 26)	85.53 (11.87)	91.46 (10.90)	5.92	2.00, 9.84	3.11 **	0.60
Psychological Distress (*n* = 26)	23.50 (9.47)	17.50 (6.09)	6.00	−9.56, −2.44	−3.48 **	−0.67
Aerobic (*n* = 6)	6.22 (3.15)	6.20 (2.87)	0.02	−1.33, 1.30	−0.03	−0.12
Weight (*n* = 14)	89.57 (12.58)	89.12 (11.39)	0.45	−1.75, 0.85	−0.75	−0.19

* *p* < 0.05, ** *p* < 0.01, *** *p* < 0.001.

**Table 6:** His Tribe Participant Personal Strengths, Relationship–community–cultural (RCC) Strengths, and Psychological Distress outcomes at Post-Program and 6-month Follow up.

	His Tribe Post-Program and 6-Month Follow up Comparisons					
	Pre *M* (SD)	Post *M* (SD)	Mean Diff	95% CI	t	Cohen’s d
Personal Strengths (*n* = 10)	134.10 (25.82)	130.30 (12.18)	3.80	−17.0, 9.47	−0.65	−0.20
RCC Strengths (*n* = 10)	92.40 (10.23)	91.40 (10.61)	1.0	−5.91, 3.91	−0.46	−0.14
Psychological Distress (*n* = 10)	18.90 (5.16)	17.70 (4.85)	1.20	−2.01, 4.41	−0.85	−0.26

* *p* < 0.05, ** *p* < 0.01, *** *p* < 0.001.

**Table 7:** Core themes of Individual and Relationship changes and quotes from participants.

Individual Changes	Quotes: Her Tribe Participants (3 Yarning Circles–YC1, 2, 3)	Quotes: His Tribe Participants (1Yarning Group: 10 Participants)
Personal strengths and skills	How many times did I say “I’m not doing that, I can’t do that”? and then I went and did it (YC1)	I’ve got more energy that in my twenties, like a lot more energy and mentally as well, I feel a lot better (P4)
Lifestyle changes	I think the biggest thing was the education as well. Now I don’t eat the Macas. I have the understanding of what it does, sort of thing, or sugary drinks (YC2)	I maintained sort of a fitness regime after the program (P3). Same here but I still gotta’ get the nutrition right (P1)
Mental health and SEWB	It really made me think about my mental health as well, a lot with the pre-talks before the exercise. And really inspired me to change my mental and physical health (P4) It was like holistic I think, and that was the big thing for me because there’s being physically fit but for me mentally fit was just as, if not more, important (P13)	I’ve had several issues with mental health…and that really… to realise that you’re not the only one out there (P10) It was good always meeting…a really positive thing, it was rare for me that I got depressed in-between that time I was coming (P5)
Cultural identity and connection	They were threatening to suspend me (from another program) because I was ‘too white’ to be Aboriginal. I had so much shame after that… now it’s like I’ve got community, that’s big’ (YC2)	Not just coming together as men, but just spiritually healing for everyone, whether you were hurting or not (P7).
Relationship Changes	Quotes: Her Tribe participants (3 Yarning Circles–YC1, 2, 3)	Quotes: His Tribe participants (1Yarning Group: 10 participants)
Community connection	I also made a lot of friendships out of it, like people that I see in community now that I never knew before, but I see them all the time now at events (P4)	I thought it was a good place for connection. I don’t think men connect enough. So, ah, to have more spaces where we can create that sort of environment (P6)
Safety	I felt in a safe environment and for me that safety was…. it was an amazing part of it. Just being accepted (YC2)	Not made to feel unwelcome. A lot of times we all feel unwelcome, no matter where we go as men (P8)
Inspiration and support	It was really good to have other people around, and they’re there supporting the achievement you were making, but [I] didn’t realise they were there supporting me through other things as well (YC)	We grew as a group of men, help each other with each other’s problems… like us men, we usually [do, we] avoid the issue (P8)
Role models for children	I kept coming back because I loved it and it was good that I could bring my kids and then show them, be a good role model for them (P1)	We get to meet each other’s little kids… it brings everyone a bit closer… the kids see that person and are like “I know you!” (P6)

**Table 8:** Themes from experiences of program structure and content and participant feedback about the Aboriginal Resilience and Recovery Questionnaire.

Program Structure and Content	Quotes: Her Tribe Participants (3 Yarning Circles–YC1, 2, 3)	Quotes: His Tribe Participants (1Yarning Group: 10 Participants)
Holistic design	I like the way it was run, have the speakers come in and then go out, and do the work out (YC1)	We all helped each other and to see some of the Elders….they’re leaders…and you look over at Uncle pumping it out, and you’re like “I better not slacken off!” (P10)
Improvements	Make it a normal thing every week (P8)	One thing I didn’t like is turn up and have a feed and then you sit down and talk ….. you’ve had the feed, settled, then to try and get up and to do the exercise. I think exercise first and then the meals (P4)
Post-program loss	It was by far the best thing in my week. There was a post Her Tribe depression. It wasn’t like that extreme but it was like you really longed for it… that’s what made it so important, belonging..’(YC2)	Shortly after it… [the program finishing] I could feel that, like, my mental health dropping (P3) I felt the drop [in mental health] after (P6).
Research acceptable	At the start I was a bit like, not iffy, but it was a bit like “oh ok, so what parts and all that?”… but that quickly got away. I sort of came to realise it was going to be different. It actually turned from “oh how are you going to use this? to empowering because you’re actually a part of it sort of thing”.	No problems (P1)
Participant feedback	ARRQ Feedback Her Tribe participants	ARRQ feedback His Tribe participants
	When I did it today, it was like, 80% of it was over the right-hand side [positive scores] rather than the left and that was a bit, I was like “wow, things have changed a bit”. (YC2) Made me think about the positives that I don’t often stop and think about. Completing this questionnaire makes me realise how resilient I am and how strong I am in my identity, culture and values’. (ARRQ written feedback) The questionnaire helped me reflect and think about how I have overcome adversity and how much I need to strengthen my connection to country and mob for my own wellbeing. (ARRQ written feedback)	It’s a good measurement tool… before and after... indicates what value the program was (P7) Participating in His Tribe positively impacted on my wellbeing. The questionnaire reflects these results. (ARRQ written feedback) The questionnaire gave me an opportunity to reflect on my life and identify issues in my personal life and in the community. (ARRQ written feedback)

**Table 9:** Her Tribe Participant Post-Hoc Analysis of Safety, Community Connection, Positive Emotions, and Self-Worth Subscales.

Outcomes	Her Tribe Pre- and Post-Program Sub-Scale Comparisons					
	Pre M (SD)	Post M (SD)	Mean Diff	95% CI	t	Cohen’s d
Safety (*n* = 43)	20.53 (4.13)	21.93 (3.84)	1.40	2.47, 0.32	2.62 **	3.56
Community connection (*n* = 44)	16.57 (2.68)	17.83 (2.19)	1.27	2.06, 0.48	3.25 ***	2.64
Positive Emotions (*n* = 45)	15.69 (3.22)	17.06 (2.83)	1.38	2.28, 0.47	3.07 **	3.06
Self-Worth (*n* = 44)	7.45 (1.82)	8.27 (1.83)	0.80	1.34, 0.30	3.16 ***	1.74

* *p* < 0.05, ** *p* < 0.01, *** *p* < 0.001.

**Table 10:** His Tribe Participant Post-Hoc Analysis of Safety, Community Connection, Positive Emotions, and Self-Worth Subscales.

Outcomes	His Tribe Pre- and Post-Program Sub-Scale Comparisons					
	Pre M (SD)	Post M (SD)	Mean Diff	95% CI	t	Cohen’s d
Safety (*n* = 26)	19.27 (3.97)	21.65 (3.46)	2.38	3.76, 1.00	3.57 ***	3.51
Community connection (*n* = 26)	17.23 (2.77)	18.38 (2.02)	1.15	1.91, 0.39	3.11 **	1.95
Positive emotions (*n* = 26)	12.04 (1.77)	17.23 (2.41)	5.19	6.14, 4.24	11.27 ***	2.42
Self-worth (*n* = 44)	7.65 (1.41)	8.65 (1.35)	1.67	1.68, 0.32	3.05 **	1.73

* *p* < 0.05, ** *p* < 0.01, *** *p* < 0.001.

## Data Availability

De-identified data supporting reported results of this study can be obtained from first author G.G. upon request.

## Appendix A

The 10 item Kessler psychological distress scale questions that were used in the programs are listed below. Further information about the measure and scoring can be found in the following paper: Kessler, R. C., Andrews, G., Colpe, L. J., Hiripi, E., Mroczek, D. K., Normand, S. L., … & Zaslavsky, A. M. (2002). Short screening scales to monitor population prevalences and trends in non-specific psychological distress. *Psychological medicine*, *32*(6), 959–976 [14].

The K10 psychological distress scale questions:

1. During the last 30 days, about how often did you feel tired out for no good reason?
2. During the last 30 days, about how often did you feel nervous?
3. During the last 30 days, about how often did you feel so nervous that nothing could calm you down?
4. During the last 30 days, about how often did you feel hopeless?
5. During the last 30 days, about how often did you feel restless or fidgety?
6. During the last 30 days, about how often did you feel so restless you could not sit still?
7. During the last 30 days, about how often did you feel depressed?
8. During the last 30 days, about how often did you feel that everything was an effort?
9. During the last 30 days, about how often did you feel so sad that nothing could cheer you up?
10. During the last 30 days, about how often did you feel worthless?
